# Protein Kinase A-Mediated Septin7 Phosphorylation Disrupts Septin Filaments and Ciliogenesis

**DOI:** 10.3390/cells10020361

**Published:** 2021-02-09

**Authors:** Han-Yu Wang, Chun-Hsiang Lin, Yi-Ru Shen, Ting-Yu Chen, Chia-Yih Wang, Pao-Lin Kuo

**Affiliations:** 1 Department of Obstetrics and Gynecology, College of Medicine, National Cheng Kung University, Tainan 701, Taiwan; S58071085@gs.ncku.edu.tw (H.-Y.W.); j0922615009@yahoo.com.tw (C.-H.L.); yanyup2003@gmail.com (Y.-R.S.); 2 Institute of Basic Medical Sciences, College of Medicine, National Cheng Kung University, Tainan 701, Taiwan; qooq118@hotmail.com; 3 Department of Cell Biology and Anatomy, College of Medicine, National Cheng Kung University, Tainan 701, Taiwan; 4 Department of Obstetrics and Gynecology, National Cheng-Kung University Hospital, Tainan 704, Taiwan

**Keywords:** septin7, septin filament, primary cilium, protein kinase A, catalytic subunit

## Abstract

Septins are GTP-binding proteins that form heteromeric filaments for proper cell growth and migration. Among the septins, septin7 (SEPT7) is an important component of all septin filaments. Here we show that protein kinase A (PKA) phosphorylates SEPT7 at Thr197, thus disrupting septin filament dynamics and ciliogenesis. The Thr197 residue of SEPT7, a PKA phosphorylating site, was conserved among different species. Treatment with cAMP or overexpression of PKA catalytic subunit (PKACA2) induced SEPT7 phosphorylation, followed by disruption of septin filament formation. Constitutive phosphorylation of SEPT7 at Thr197 reduced SEPT7‒SEPT7 interaction, but did not affect SEPT7‒SEPT6‒SEPT2 or SEPT4 interaction. Moreover, we noted that SEPT7 interacted with PKACA2 via its GTP-binding domain. Furthermore, PKA-mediated SEPT7 phosphorylation disrupted primary cilia formation. Thus, our data uncover the novel biological function of SEPT7 phosphorylation in septin filament polymerization and primary cilia formation.

## 1. Introduction

Septins are GTP-binding proteins that polymerize into heterooligomeric core complexes, and further assemble into higher-order structures, including filaments, rings, and cages [1]. The septin filaments play important roles in several biological processes in mammalian cells, including cytokinesis, plasma membrane dynamics, and ciliogenesis [2,3,4,5,6]. All septins consist of an amino and carboxyl terminus, called the N-terminus and C-terminus, and a guanine nucleotide binding site, called the GTP-binding domain (GBD) [7]. Based on the homology of sequences, the mammalian septins are classified into four subgroups: the SEPT2 subgroup (SEPT1, SEPT2, SEPT4, and SEPT5), the SEPT3 subgroup (SEPT3, SEPT9, and SEPT12), the SEPT6 subgroup (SEPT6, SEPT8, SEPT10, SEPT11, and SEPT14), and the SEPT7 subgroup (SEPT7 only) [8,9,10].

SEPT7 plays important roles during cell division, cytokinesis, and neuronal differentiation. For example, depletion of SEPT7 leads to microtubule destabilization as well as cytokinesis defects in fibroblasts [11]. SEPT7 is also required for chromosome alignment, affecting the extrusion of the second polar body during meiosis [12]. In mature neurons, depletion of SEPT7 results in decreasing dendritic branching and dendritic spine morphogenesis [13]. Thus, precise regulation of SEPT7 is crucial for development and differentiation.

SEPT7 is a key component of most types of septin filaments [8]. Septins can hetero-oligomerize into the hexamer core complex SEPT2‒SEPT6‒SEPT7‒SEPT7‒SEPT6‒SEPT2. SEPT7 interacts with SEPT6 and SEPT7 via the N‒C termini and GBD, respectively [14]. Therefore, SEPT7 assembles several septin members into a multimeric structure to carry out its biological functions.

Phosphorylation is an important mechanism in the regulation of septin function. During the epididymal transition of spermatozoa, defective SEPT4 phosphorylation results in a defective membrane diffusion barrier [15]. In the brain, SEPT4 phosphorylation is regulated by the dual-specificity tyrosine phosphorylation-regulated kinase 1A (DYRK1A) [16]. The phosphorylation of SEPT3 by PKG modulates neuronal function [17]. PLK1 interacts with and phosphorylates SEPT9, thus affecting cytokinesis [18]. Therefore, the phosphorylation of septins is important for various physiological functions.

The primary cilium is a microtubule-based organelle that protrudes from the surface of most mammalian cells. It plays important roles in development and differentiation. Disruption of cilium structure or function causes many diseases, including obesity, blindness, polycystic kidney disease, mental retardation, and polydactyly [19]. The primary cilium is composed of a central axoneme, which is the extended structure of the mother centriole, and an overlying ciliary membrane [20,21,22]. Septins have been shown to localize to the primary cilium and are involved in the formation of cilia, known as ciliogenesis [23]. For example, SEPT2 functions as diffusion barriers at the base of the ciliary membrane; the loss of ciliary membrane protein localization and inhibited ciliogenesis are observed in SEPT2-deficient cells [22]. In *Xenopus* embryos, knockdown of SEPT7 or SEPT2 causes defects in ciliogenesis [23]. In mammals, the SEPT2‒SEPT7‒SEPT9 complex localizes to the axoneme of cilia for ciliogenesis and the maintenance of ciliary length [21]. Thus, septin complexes are required for maintaining cilia growth and length [24,25,26].

In this study, we aimed to explore whether SEPT7 phosphorylation influences septin filament assembly and ciliogenesis. We found that PKA increased SEPT7 phosphorylation at the Thr. Overexpression of phospho-memetic SEPT7 reduced filament assembly, suggesting that SEPT7 phosphorylation at Thr197 is important for the assembly of SEPT7 filaments. We also found that PKA interacted with SEPT7 through the GTP-binding domain. Furthermore, we found that PKA-mediated phosphorylation of SEPT7 reduced the frequency of ciliated cells and reduced the ciliary length. These findings suggest that SEPT7 phosphorylation disrupts septin filament assembly and ciliogenesis.

## 2. Materials and Methods

### 2.1. DNA Constructs, Cell Culture, and Transfection

Human SEPT7 and PKA were amplified from a human RNA panel (Clontech, Mountain View, CA, USA) and cloned into pFLAG-CMV-2, pEGFP-C3, and pCDNA3-HA vectors, as described previously [27]. All constructs were verified by DNA sequencing. For transient transfection, malignant human testis pluripotent embryonic carcinoma NT2/D1 cells, REP cells, and human embryonic kidney 293 T cells were incubated in Dulbecco’s minimal essential medium (DMEM), supplemented with 10% fetal bovine serum (FBS) and 1% antibiotics. Cells were transfected with plasmids using Lipofectamine 2000 (Thermo Fisher Scientific, Waltham, MA, USA) according to the manufacturer’s instructions. After 24 h, the cells were subjected to immunofluorescence staining or immunoblotting. Alternatively, cells were treated with 8-bromo-cAMP or Na_3_VO_3_ (Sigma-Aldrich, Darmstadt, Germany) as indicated for analysis of protein expression.

### 2.2. Immunoprecipitation Assay and Western Blot Analysis

For immunoprecipitation analysis, 2 μg of indicated antibodies was incubated with Dynabeads Protein G (Thermo Fisher Scientific) at room temperature for 15 min on a rotator, and the cell lysates were immunoprecipitated with the bead‒antibody complex at 4 °C overnight on a rotator. The samples were washed three times with a wash buffer (100 mM Tris, 150 mM NaCl, 2 mM EDTA, 0.5% Tween-20, and 0.01% NP-40). The precipitates were mixed with SDS sample buffer, then boiled for 10 min. For Western blotting, the proteins were adjusted to an equal amount of protein, electrophoresed through sodium dodecyl sulfate-polyacrylamide gel electrophoresis (SDS-PAGE), and blotted onto PVDF membranes (Merck Millipore, Darmstadt, Germany). The blots were then incubated with an anti-FLAG (1:5000; Sigma-Aldrich, F1804, SICC6485), anti-Myc (1:5000; GeneTex, Irvine, CA, USA, GTX115046, 40863), anti-GFP (1:4000; GeneTex, GTX113617, 42060), anti-HA (1:5000; BioLegend, San Diego, CA, USA, 901514, B224726), anti-PKA (1:1000, BD, Franklin Lakes, NJ, USA, 610980) antibody, anti-SEPT4 (1:1000, Sigma-Aldrich, A4471, 106M4840), anti-SEPT6 (1:1000, Santa Cruz Biotechnology, Dallas, TX, USA, SC20180, A2030), anti-SEPT7 (1:1000, Sigma-Aldrich, HPA029524, B117734), or antiphosphothreonine (1:1000, Cell Signaling, Danvers, MA, USA, 93863, 10) antibody. Immunofluorescence analysis of filament formation followed.

### 2.3. Immunofluorescence

For the immunofluorescence analysis, the plasmids were transfected into NT2/D1 cells or 293T cells using Lipofectamine 2000 (Thermo Fisher Scientific). After 24 h, NT2/D1 cells were fixed with 4% paraformaldehyde in phosphate-buffered saline (PBS) permeabilized with 0.1% triton X-100 in PBS, and blocked with the antibody diluent (Dako North America, Santa Clara, CA, USA) for 1 h. NT2/D1 cells or 293T cells were washed three times with PBS. The filament could then be visualized using fluorescence microscopy (Olympus, Tokyo, Japan, BX60).

### 2.4. ClustalW multiple Sequence Alignment

The human SEPT7 orthologous proteins were used for multiple sequence alignment by the ClustalW2 program (http://www.ebi.ac.uk/, accessed on 25 January 2020). The accession numbers for SEPT7 proteins of different species were as follows: *Homo sapiens* (NP_001779.3 isoform 1, NP_001011553.2 isoform 2), *Sus scrofa* (pig) (XP_003134838.1 isoform X1), *Canis lupus familiaris* (dog) (XP_022283282.1), *Felis catus* (domestic cat) (XP_003982994.1), *Mus musculus* (house mouse) (NP_033989.2), *Rattus norvegicus* (brown rat) (NP_072138.2), *Bos taurus* (cattle) (NP_001001168.1), *Pongo abelii* (Sumatran orangutan) (NP_001126872.1), *Xenopus laevis* (African clawed frog) (NP_001086183.1), *Xenopus tropicalis* (tropical clawed frog) (XP_002939459.1), and *Danio rerio* (zebrafish) (NP_001242958.1). The accession numbers for SEPTIN groups of *Homo sapiens* were as follows: SEPT1 (NP_443070.1), SEPT2 (NP_001008491.1), SEPT3 (isoform A: NP_663786.2; isoform B: NP_061979.3), SEPT4 (isoform 1: NP_004565.1; isoform 2: NP_536340.1; isoform 3: NP_536341.1), SEPT5 (NP_002679.2), SEPT6 (isoform A: NP_665798.1; isoform B: NP_055944.2; isoform D: NP_665801.1), SEPT7 (isoform 1: NP_001779.3; isoform 2: NP_001011553.2), SEPT8 (isoform A: NP_001092281.1; isoform B: NP_055961.1; isoform C: NP_001092282.1; isoform D: NP_001092283.1), SEPT9 (isoform A: NP_001106963.1; isoform B: NP_001106965.1; isoform C: NP_006631.2; isoform D: NP_001106967.1; isoform E: NP_001106964.1; isoform F: NP_001106968.1), SEPT10 (isoform 1: NP_653311.1; isoform 2: NP_848699.1), SEPT11 (NP_060713.1), and SEPT14 (NP_997249.2). The above information was provided by the National Center for Biotechnology Information (NCBI) database (http://www.ncbi.nlm.nih.gov/, accessed on 25 January 2020).

### 2.5. Examining Primary Cilia Formation

Wild-type RPE1 cells were grown on glass cover slips at 37 °C overnight. For SEPT7-deficient RPE1 cells, SEPT7 was depleted by transfecting RPE1 cells with siRNA against SEPT7 for 3 days. Then, cells were cultured in a serum-free medium for 24 h for inducing primary cilia formation. All cells were fixed with ice-cold methanol at −20 °C for 6 min. After blocking with 5% BSA for 1 h, the cells were incubated with primary antibodies for 24 h at 4 °C. After extensive washing with PBS, the cells were incubated with fluorescein isothiocyanate- and Cy3-conjugated secondary antibodies (Invitrogen, Carlsbad, CA, USA, A10521) for 1 h in the dark. The nuclei were stained simultaneously with 4′,6-diamidino-2-phenylindole (DAPI, 0.1 μg/mL). After extensive washing, the cover slips were mounted on glass slides in 50% glycerol. Primary cilia were imaged with an Axio Imager M2 fluorescence microscope (Zeiss, Oberkochen, Germany) and captured using ZEN Pro software (Zeiss). Primary cilia images were generated, and the length of cilia was measured from z-stacks using add-on features of the ZEN Pro software.

### 2.6. Statistical Analysis

Data were expressed as the mean ± SEM. Statistical significance was determined by one-way analysis of variance (ANOVA), combined with Tukey’s multiple comparison test for posterior comparisons. *p*-values were considered significant at * *p* < 0.05; ** *p* < 0.01; and *** *p* < 0.001.

## 3. Results

### 3.1. Conservation of the SEPT7 Phosphorylation Site among Species

**Figure 1:**
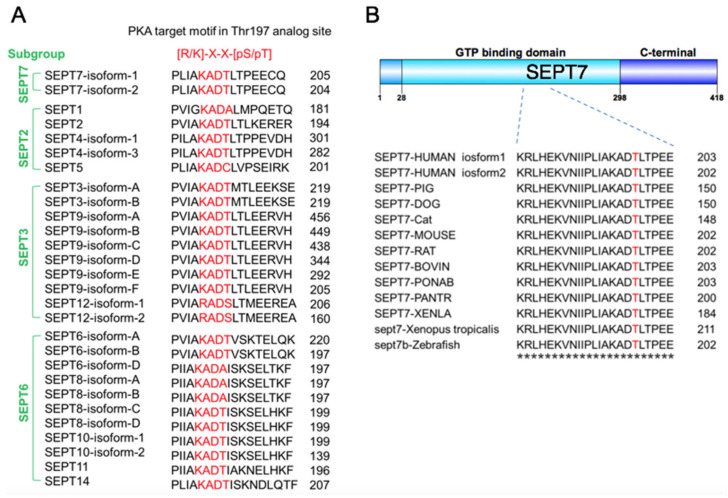
Conservation of PKA target SEPT7 phosphorylation site Thr197 among species. (**A**) Multiple sequence alignment flanking the analogous Thr197 residue of SEPT7 in 13 different human genes coding for septins. Alignment of the sequences is according to the PKA consensus target motif [R/K]-X-X-[pS/T]. (**B**) Multiple sequence alignment of analogous sites of Thr197 in the SEPT7 orthologues and comparison of the amino acids in various species. Red indicates that the residues’ alignment was similar. At the bottom, the asterisk denotes an identical residue in all sequences in the alignment. The amino acid sequences were analyzed using the ClustalW2 program at EMBL-EBI.

Our previous study showed that PKA phosphorylates SEPT12 at Ser196, which is located within the GTP-binding domain (GBD), thus disrupting septin filaments in the sperm annulus [28]. GBD is conserved in the septin family, so thus screened the PKA recognition motif, [R/K]-X-X-[pS/T], using amino acid alignments of 28 septin sequences selected from all septin subgroups. This PKA recognition motif was identified in most septins, including SEPT2, SEPT3, SEPT4, SEPT7, SEPT9, SEPT10, SEPT11, and SEPT14 (Figure 1A). Here, we were interested in SEPT7 as it was the irreplaceable component of all septin filaments [7]. The putative PKA phosphorylation site of human SEPT7 was located on the Thr197, so we checked whether this PKA phosphorylation site was conserved among different species. From zebrafish to *Homo sapiens*, the predicted phosphorylation site is highly conserved (Figure 1B). Thus, Thr197 of SEPT7 was a putative phosphorylation site targeted by PKA.

### 3.2. SEPT7 Is Phosphorylated by PKA

**Figure 2:**
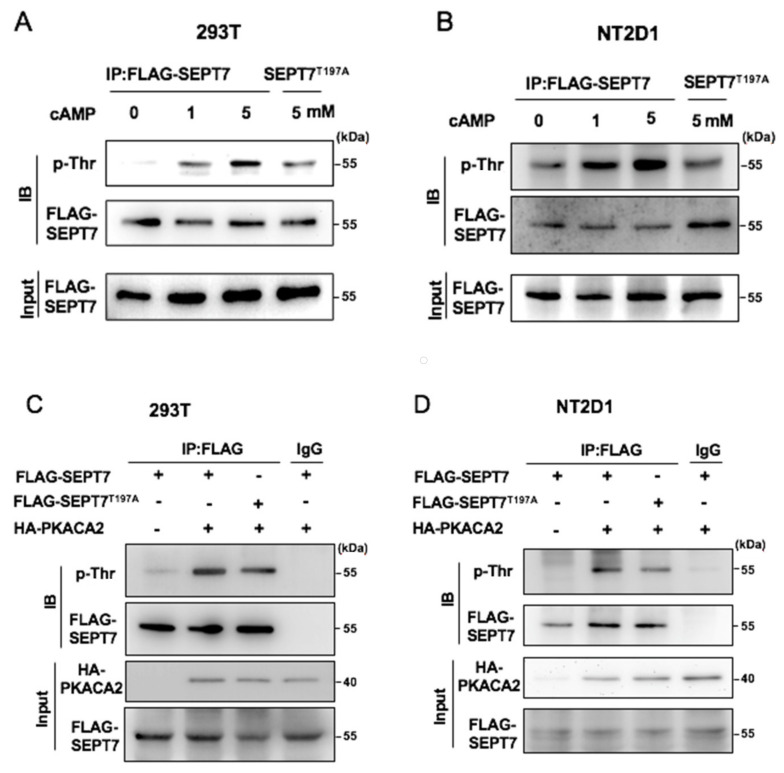
SEPT7 was phosphorylated at the Thr197 site by PKA. Activation of PKA increased SEPT7 phosphorylation at the Thr197 site following treatment with 8-Br-cAMP in a dose-dependent manner, but was reduced in a phosphodeficient SEPT7 (T197A) mutant of 293T (**A**) or NT2/D1 (**B**) cells. (**A**) FLAG-SEPT7 or FLAG-SEPT7 (T197A) was transfected into 293T cells following treatment with 0, 1, or 5 mM 8-Br-cAMP. The levels of the Thr197 phosphorylation were increased in FLAG-SEPT7-transfected 293T cells. Extracts of FLAG-SEPT7-transfected 293T cells or FLAG-SEPT7 (T197A) mutant 293T cells following treatment with 8-Br-cAMP were analyzed by immunoprecipitation (IP) with antibodies against FLAG. (**B**) FLAG-SEPT7 or FLAG-SEPT7 (T197A) mutant was transfected into NT2/D1 cells. The levels of Thr phosphorylation were increased in FLAGSEPT7-transfected NT2/D1 cells, but reduced in FLAG-SEPT7 (T197A) mutant NT2/D1 cells, following treatment with 8-Br-cAMP. Extracts of FLAG-SEPT7 or FLAG-SEPT7 (T197A) mutant-transfected NT2/D1 cells, following treatment with 8-BrcAMP, were analyzed by immunoprecipitation with antibodies against FLAG. PKA phosphorylates SEPT7 on Thr. (**C**,**D**) FLAG-SEPT7 and FLAG- SEPT7 (197A) mutant, with or without HA-PKACA2, were transfected into 293T cells (**C**) and NT2/D cells (**D**) as indicated, and the expression of phosphorylated threonine, SEPT7, and PKACA2 was detected with antibodies against phospho-threonine, FLAG, and HA. IgG served as a negative control.

Next, we checked whether PKA was responsible for SEPT7 phosphorylation at Thr197. Due to lacking a specific antibody against phosphorylated SEPT7 at Thr197, FLAG-tagged SEPT7 was transfected into cells, and cell lysates were immunoprecipitated with a FLAG antibody. The immunoprecipitants were analyzed by an immunoblotting assay with an antibody against phosphothreonine. Treatment with 8-Br-cAMP, the activator of PKA, increased the phosphorylation on Thr sites of SEPT7 in 293T (Figure 2A) and NT2/D1 (Figure 2B) in a dose-dependent manner. Importantly, this phosphorylation signal was reduced in a phosphodeficient SEPT7 (T197A) mutant. These results suggest that the activation of PKA increased SEPT7 phosphorylation at the Thr197 site. To further confirm this finding, the catalytic subunit of PKA, PKACA2 (encoded by the gene *PRKACA*), was co-transfected with wild-type SEPT7 or T197A mutant followed by an examination of the phosphorylation status of SEPT7. Overexpression of PKACA2 increased the Thr phosphorylation of SEPT7, and this signal was reduced in the T197A mutant of the 293T and NT2/D1 cell lines (Figure 2C,D). Thus, PKA phosphorylates SEPT7 on Thr197.

### 3.3. SEPT7 Interacts with PKA via the GTP-Binding Domain

**Figure 3:**
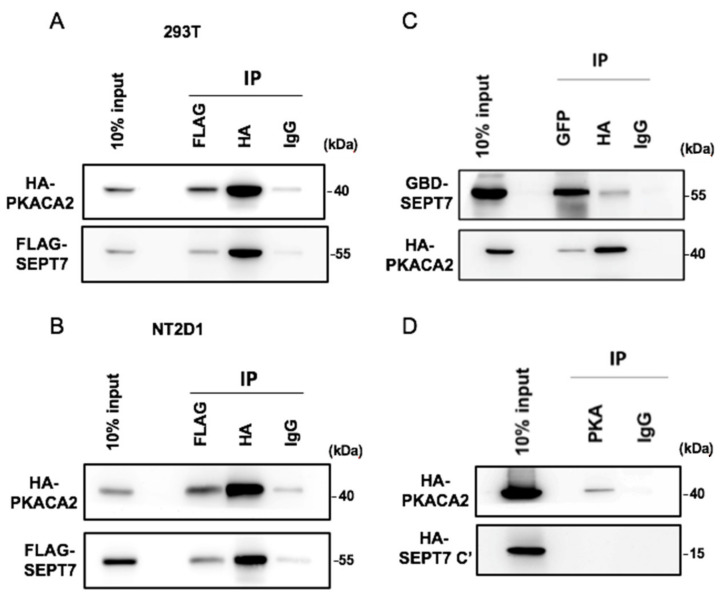
SEPT7 interacts with PKA via the GTP-binding domain. SEPT7 interacted with PKA (**A**,**B**) via its GTP-binding domain, not its C-terminus (C’) domain (**C**). FLAG-SEPT7 and HA-PKACA2 were co-transfected to 293T cells or NT2/D1 cells followed by an immunoprecipitation assay. (**A**,**B**) PKACA2 was detected in the precipitant of FLAG-SEPT7 and vice versa. Extracts of FLAG-SEPT7 and HA-PKACA2 co-transfected 293T or NT2/D1 cells were immunoprecipitated by FLAG or HA with antibodies against HA and FLAG. Transfected IgG served as a negative control. Lanes showing 10% of the input are also present. (**C**) GFP-tagged GBD of SEPT7 (GFP-GBD-SEPT7) and HA-PKACA2 were co-transfected to NT2/D1 cells, followed by an immunoprecipitation assay. Extracts of GFP-GBD-SEPT7 and HA-PKACA2 co-transfected NT2/D1 cells were immunoprecipitated by GFP or HA with antibodies against HA and GFP. (**D**) Co-transfected HA-SEPT7-C terminal and HA-PKACA2 in NT2/D1 cells, followed by the performance of an immunoprecipitation assay. Extracts of HA-SEPT7 C’ and HA-PKACA2 co-transfected NT2/D1 cells were immunoprecipitated by PKA with an antibody against PKA. Transfected IgG served as a negative control. Lanes showing 10% of the input are also present.

We then checked whether SEPT7 interacted with PKACA2. FLAG-tagged SEPT7 and HA-tagged PKACA2 were co-transfected into NT2/D1 cell lines, followed by an immunoprecipitation assay. PKACA2 was detected in the precipitant of FLAG-SEPT7 and vice versa (Figure 3A,B), suggesting that SEPT7 interacted with PKACA2. Next, the interacting domain of SEPT7 was examined. SEPT7 was dissected into the N-terminus (N’), GTP-binding domain (GBD), and C-terminus (C’). Unfortunately, the expression of N’ of SEPT7 was unstable, so it could not be used for immunoprecipitation assay. We then examined whether the GBD or C’ of SEPT7 interacted with PKACA2. GFP-tagged GBD or C’ was co-transfected with HA-tagged PKACA2 into NT2/D1 cell lines, followed by an immunoprecipitation assay. PKACA2 was detected in the precipitant of GFP-tagged GBD (Figure 3C), but not in the C’ precipitant (Figure 3D), and vice versa. Thus, SEPT7 interacts with PKACA2 via the GBD.

### 3.4. SEPT7 Phosphorylation Disrupts Septin Filament Formation

**Figure 4:**
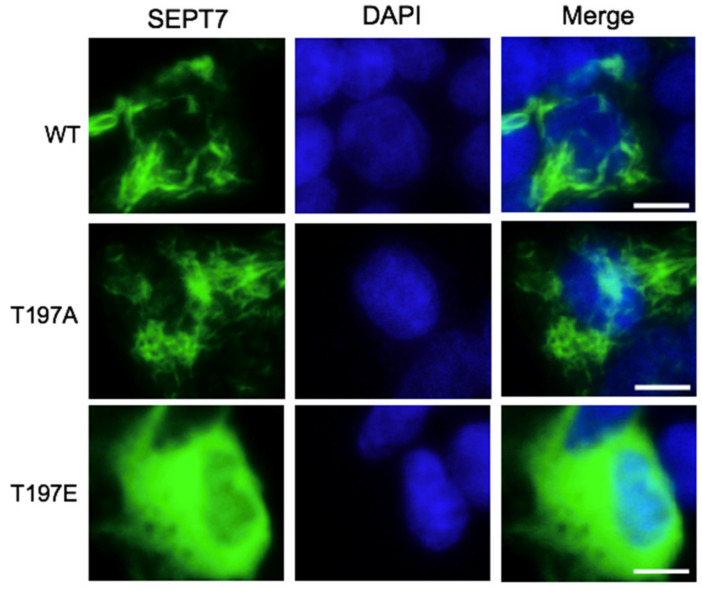
SEPT7 phosphorylation disrupts septin filament formation. The plasmids of GFP-SEPT7, GFP-SEPT7 T197A (de-phosphorylated status), and GFP-SEPT7 T197E (constitutive phosphorylated status) were transfected into 293T cells. Septin filaments were observed in cells transfected with wild-type (WT) SEPT7 (upper panel) and T197A (middle panel), but not in T197E (lower panel) constructs. DNA was stained with DAPI (blue). Scale bar: 10 μm (white bar).

**Figure 5:**
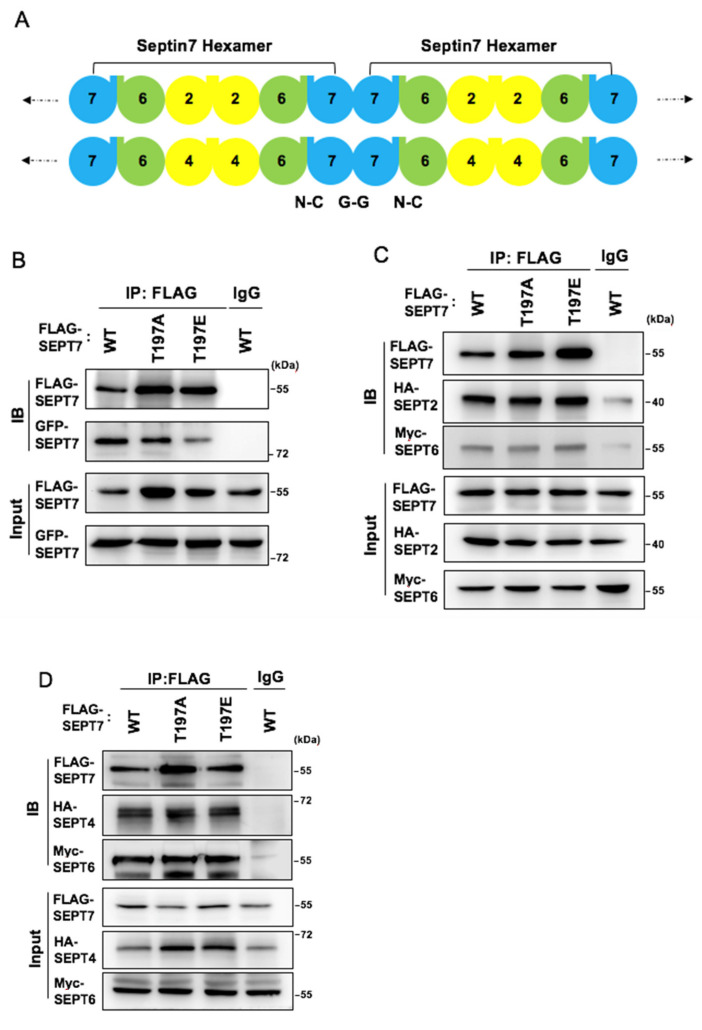
Mimetic phosphorylated Thr197 of SEPT7 disrupts SEPT 7-7 complex but does not affect SEPT 2, SEPT 4, and SEPT 6 interaction. (**A**) Schematic representation of a heteromeric SEPT7–SEPT6–SEPT2 or SEPT7–SEPT6–SEPT4 complex. SEPT7 has been included at the terminal positions (shown in blue). The SEPT 7‒SEPT 7 pairwise interactions occur at the G-interface, whilst the SEPT 7‒SEPT 6 dimer is stabilized by the NC interface. SEPT7 phosphorylation affected SEPT7‒SEPT7 interaction (**B**) but not SEPT 2, SEPT 4, or SEPT 6 interaction (**C**,**D**). (**B**) FLAG-SEPT7 and GFP-SEPT7 were co-transfected into NT2/D1 cells. Lysates from transiently transfected cells were immunoprecipitated by FLAG or GFP with antibodies against GFP and FLAG. (**C**,**D**) Myc-SEPT6, HA-SEPT4, and HA-SEPT2 with various FLAG-SEPT7 plasmids were transfected into NT2/D1 cells, and lysates were immunoprecipitated with an anti-FLAG antibody. The expression of SEPT2, 4, 6, and SEPT7 were detected by anti-HA, anti-Myc, and anti-FLAG antibodies, respectively. IgG served as the negative control.

SEPT7 is crucial for septin filament oligomerization, so we checked whether SEPT7 phosphorylation affected septin filament formation. Two human SEPT7 mutant constructs, T197E and T197A, which mimic the constitutive phosphorylated and dephosphorylated status, respectively, were generated, and their effects on septin filament polymerization were examined. The SEPT7 filaments were observed throughout the cytoplasm when cells were transfected with wild-type SEPT7 (Figure 4, upper panel). This phenotype was also observed in cells transfected with the phosphodeficient construct T197A (Figure 4, middle panel). However, when cells were transfected with the phosphomimetic construct T197E, the septin filaments were hardly detected, and a smear signal was shown throughout the cytoplasm (Figure 4, lower panel), suggesting that phosphomimetic SEPT7 did not form filaments. Thus, phosphorylation of SEPT7 disrupts septin filament formation. Septin filament was orchestrated by the order of the septin hexamer core complex, arranged as SEPT2-6-7-7-6-2 or SEPT4-6-7-7-6-4 [14] (Figure 5A). SEPT7 phosphorylation disrupted septin filaments; thus, the interactions of SEPT7 with different septins were examined.

### 3.5. SEPT7 Phosphorylation Disrupts SEPT7‒SEPT7 Interaction

Different septin constructs were transfected into NT2/D1 cells, followed by an immunoprecipitation assay. The interactions of FLAG-tagged SEPT7, including wild-type, T197A, or T197E, with Myc-tagged SEPT6, HA-tagged SEPT4, or HA-tagged SEPT2 were then examined via an immunoblotting assay. First, we checked whether the interaction between SEPT7, which was important for the polymerization of different septin complexes, was affected by SEPT7 phosphorylation. FLAG-tagged SEPT7 and GFP-tagged SEPT7 were co-transfected into cells; FLAG-SEPT7 was then precipitated by an anti-FLAG antibody, followed by an examination of GFP-SEPT7 with a Western blotting assay. GFP-SEPT7 could be detected in either the wild-type or T197A precipitant (Figure 5B). However, this interaction was detected to a lesser extent for T197E mutant (Figure 5B), suggesting that SEPT7 phosphorylation impeded SEPT7‒SEPT7 interaction. Next, the SEPT2-6-7 and SEPT4-6-7 complexes were checked. SEPT2 and SEPT6 were detected in wild-type SEPT7, the T197A precipitant, and T197E mutants. The data suggested that SEPT7 phosphorylation did not affect the orchestration of the SEPT2-6-7 heterocomplex (Figure 5C). SEPT7 phosphorylation status also did not affect the SEPT4-6-7 heterocomplex (Figure 5D), suggesting that SEPT7 phosphorylation affected SEPT7‒SEPT7 interaction. Our immunofluorescence data showed that overexpression of SEPT7 T197E disrupted septin filament polymerization. We also treated with phosphatase inhibitor sodium orthovanadate (Na_3_VO_4_) to understand whether phosphatase regulated SEPT7‒SEPT7 interaction. We found that Na_3_VO_4_ did not interfere with the interaction between GFP-SEPT7 and FLAG-SEPT7 (Figure S1). These data support the hypothesis that T197E disrupted the assembly of septin filament. Thus, SEPT7 phosphorylation blocks SEPT7‒SEPT7 interaction, but does not affect SEPT2-6-7 or SEPT4-6-7 complex interaction. 

### 3.6. Overexpression of PKA Disrupts SEPT7‒SEPT7 Interaction

**Figure 6:**
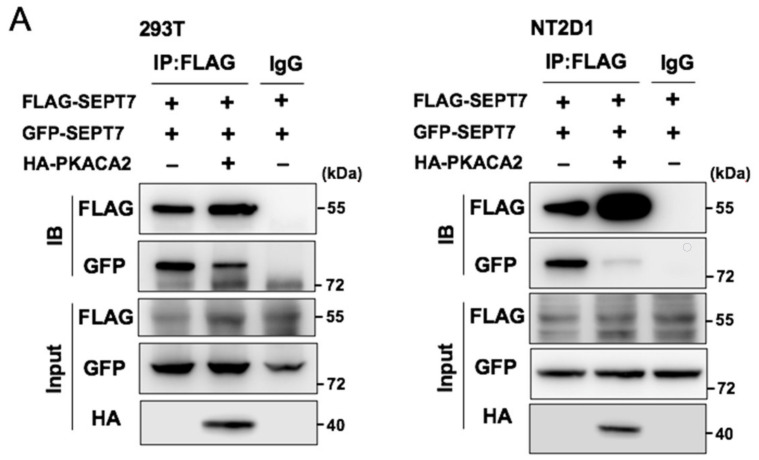

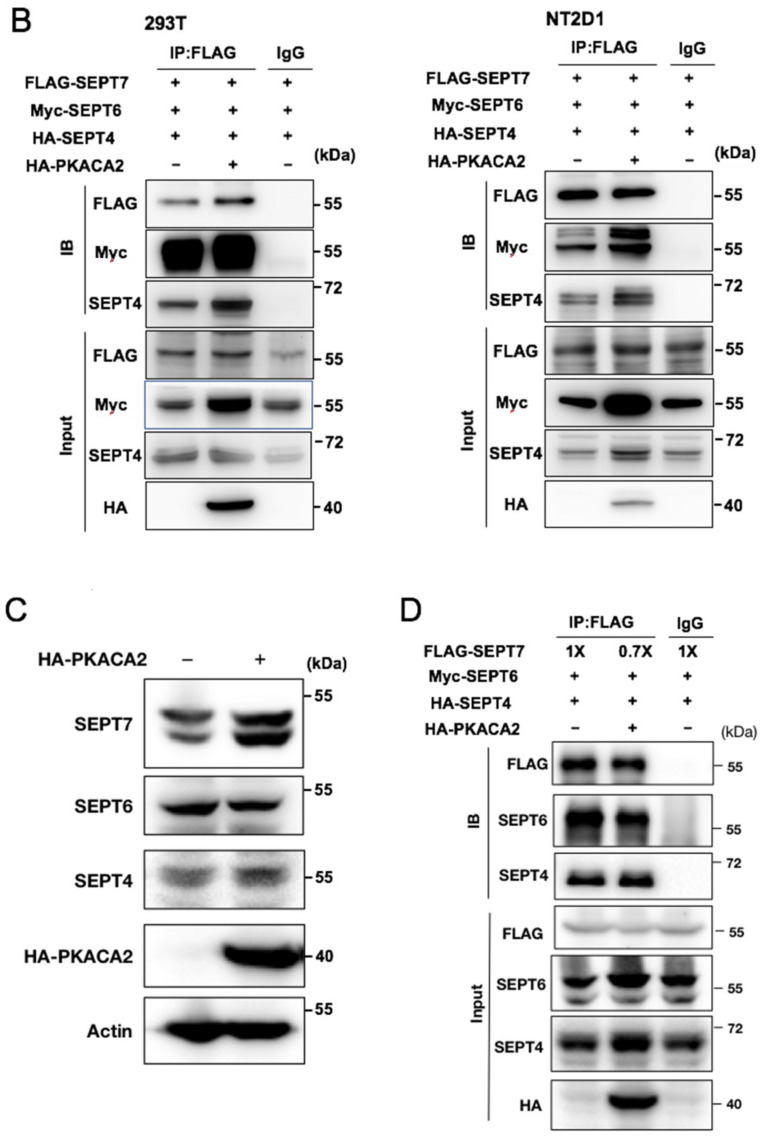
Overexpression of PKA disrupts SEPT7 filament formation. (**A**,**B**) 293T or NT2/D1 cell lines were co-transfected with FLAG-SEPT7 and GFP-SEPT7 (**A**), Myc-SEPT6 (**B**,**D**), or HA-SEPT4 (**B**,**D**), in the presence or absence of PKACA2 (**A**–**D**). Cell lysates were immunoprecipitated with an antibody against FLAG, and IgG served as a negative control. Following precipitation, samples were analyzed by immunoblotting assay with antibodies against GFP, Myc, HA, SEPT4, SEPT6, and SEPT7.

Our data showed that SEPT7 was phosphorylated by PKA, and SEPT7 phosphorylation disrupted septin filaments. Then, we checked whether overexpression of PKACA2 affected SEPT7–SEPT7 interaction. In the absence of PKACA2, SEPT7 interacted with SEPT7. However, this interaction was reduced when PKACA2 was overexpressed (Figure 6A), supporting the hypothesis that PKACA2 blocked SEPT7‒SEPT7 interaction. Then, the SEPT4‒SEPT6‒SEPT7 complexes were examined. SEPT7 interacted with SEPT6 and SEPT4, and these complexes were not affected when PKACA2 was overexpressed (Figure 6B). Thus, PKACA2 reduces SEPT7‒SEPT7 interaction, then disrupts septin filament formation. However, the expression of Myc-SEPT6 is different between the three different groups. We further determined whether PKA affected the septin expression. We found that SEPT7 expression increased after PKACA2 overexpression (Figure 6C). We further decreased the amount of SEPT7 plasmid in the PKA overexpression group. We found that the expression of Myc-SEPT6 is equal between the three different groups (Figure 6D). These data support the hypothesis that PKACA2 not only affects the SEPT7 expression but also blocks SEPT7‒SEPT7 interaction. 

### 3.7. PKA-Mediated SEPT7 Phosphorylation Affected Ciliogenesis

**Figure 7:**
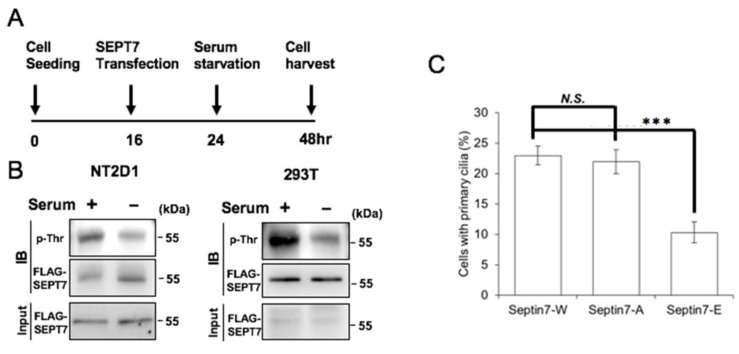

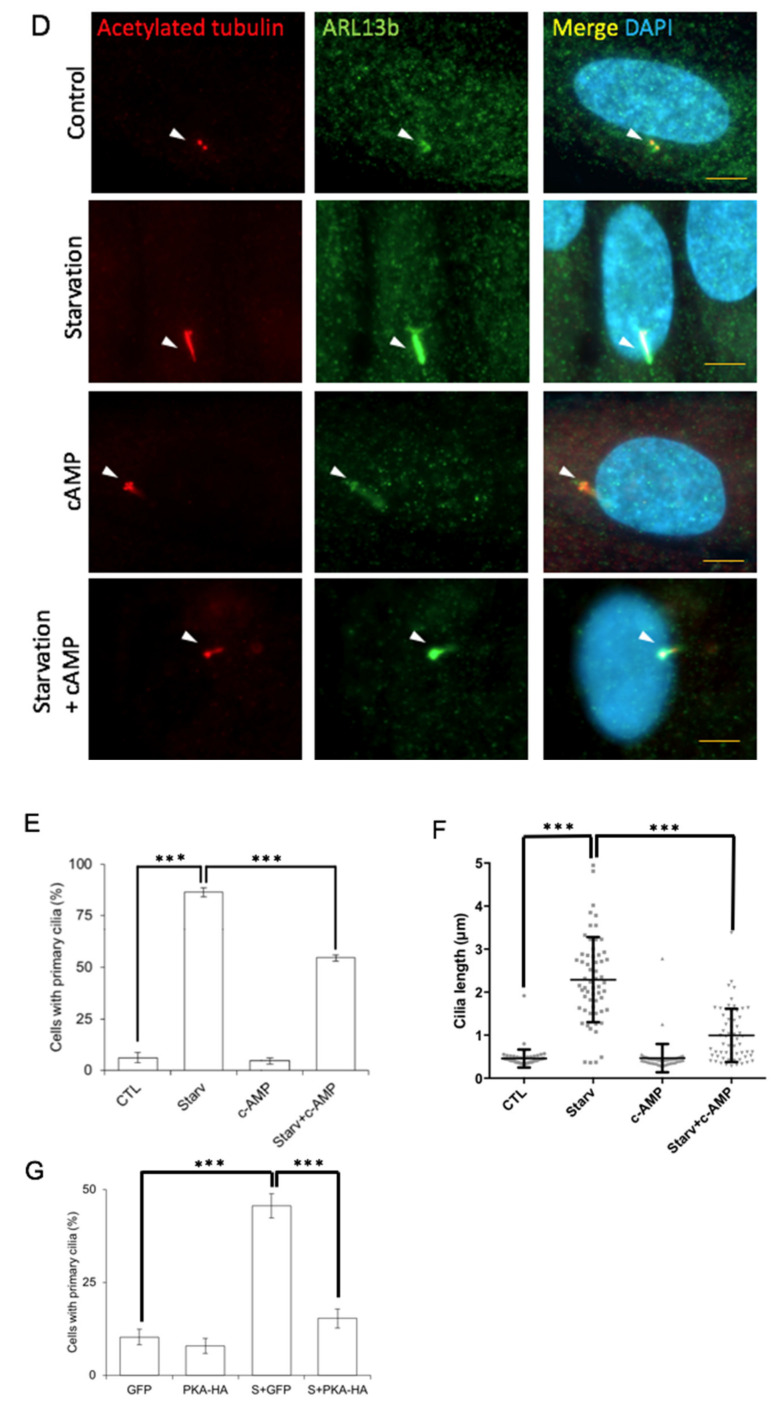
PKA-mediated SEPT7 phosphorylation affects ciliogenesis. (**A**,**B**) The level of Thr phosphorylation in 293T or NT2D1 cells was reduced after overexpression of FLAG-SEPT7 in the medium with or without serum for 24 h. Extracts of SEPT7-transfected cells with or without serum were analyzed by immunoblotting with antibodies against FLAG and phosphothreonine. Lanes showing 10% of the input are also present. SEPT7 phosphorylation inhibited primary cilia formation. (**C**) Quantitation of primary cilia in FLAG-SEPT7, FLAG-SEPT7 T197A, or FLAG-SEPT7 T197E-transfected RPE1 cells in the absence of serum. Activation of PKA followed by 8-Br-cAMP inhibited primary cilia formation (**D**,**E**) and reduced the ciliary length (**F**) under serum starvation. (**D**) Immunostaining of primary cilia and DAPI in scramble control (CTL) or 8-Br-cAMP-treated RPE1 cells. RPE1 cells were analyzed with antibodies against acetylated tubulin (ace-tub, red) and Arl13b (green). Scale bare: 5 μm. (**E**,**F**) Treatment with cAMP inhibited primary cilia formation. Quantitation of ciliated cells (**E**) or ciliary length (**F**) upon 8-Br-cAMP-treated RPE1 cells in the presence or absence of serum. (**G**) Overexpression of PKA inhibited primary cilia formation under serum starvation. RPE1 cells were transfected to GFP or HA-PKA in the presence or absence of serum (S). Quantitation of primary cilia of GFP or HA-PKA-transfected RPE1 cells under serum starvation. These results are the mean ± SD from three independent experiments; more than 100 cells were counted in each individual group. Data are shown as mean ± SEM. N.S., nonsignificant; *** *p* < 0.001

Septin-based ring filaments act as a diffusion barrier at the base of primary cilia [22]. We then investigated whether SEPT7 phosphorylation modulated primary cilia formation. Under serum starvation, cells start to grow primary cilia [29]; we thus checked whether serum starvation affected SEPT7 phosphorylation. Under serum starvation, the phosphorylation of SEPT7 was reduced (Figure 7A,B), suggesting that SEPT7 phosphorylation might affect ciliogenesis. To further confirm our hypothesis, different SEPT7 mutants were transfected into immortalized human retina pigmented epithelial (RPE1) cells, a well-established model for studying ciliogenesis, and the population of ciliated cells was quantified. Transfection of T197A had no effect on ciliogenesis when compared with transfection of wild-type SEPT7. However, the frequency of ciliation was reduced when RPE1 cells were transfected with T197E (Figure 7C). Thus, SEPT7 phosphorylation inhibits primary cilia formation. Then, the role of the cAMP-PKA cascade was examined. Treatment with cAMP reduced the frequency of ciliation and the length of cilia (Figure 7D–F). In addition, the overexpression of PKA significantly inhibited ciliogenesis (Figure 7G). Thus, SEPT7 phosphorylation inhibits ciliogenesis during serum starvation.

## 4. Discussion

In this study, we demonstrated that SEPT7 phosphorylation at Thr197 impeded SEPT7‒SEPT7 interaction but did not affect SEPT2-6-7 or SEPT4-6-7 complexes, thus inhibiting septin filament polymerization. We also demonstrated that SEPT7 phosphorylation was mediated by the cAMP–PKA axis. Furthermore, we showed that SEPT7 phosphorylation inhibited primary cilia formation and reduced cilia length. Taken together, PKA-mediated SEPT7 phosphorylation inhibits septin filament formation, thus reducing ciliogenesis. 

Post-translational modifications play an important role in the regulation of septin‒septin interactions and control the formation of a high-order septin complex. These modifications include SUMOylation, acetylation, ubiquitination, and phosphorylation. Phosphorylation of the terminal subunit septin, Cdc11, disrupted neck filaments and influenced higher-order septin architecture [30]. Phosphorylation of *Drosophila* septin Pnut (homolog of human SEPT7) in the early stages of embryogenesis disrupts the assembly of septin filament formation, thus leading to the dissociation of the septin complex. Our previous work shows that phosphorylation of a germ-cell-specific SEPT12 leads to a complete loss of the septin ring at the sperm annulus, illustrating the important roles of septin phosphorylation in the regulation of septin assembly and the formation of higher-order structures. The phosphorylation site of SEPT12 is located in the GBD, and this domain is conserved among several septins, including SEPT7. We thus checked whether phosphorylation of SEPT7 affected the assembly of the septin filament. Indeed, SEPT7 phosphorylation in the GBD led to the defective polymerization of septin filaments. Thus, phosphorylation of septin in the GBD might affect septin filament formation. However, it is still unclear whether other septins, in addition to SEPT7 and SEPT12, also show a similar phenotype, and this hypothesis still needs to be tested in the future. It has been shown that the septin complexes regulate cilium length and ciliogenesis. Ablation of any of these septins’ expression by RNA interference inhibits ciliogenesis. 

Depletion of SEPT7 results in the concomitant loss of SEPT2 and SEPT9 expression of the complex [22,31]. In the *Xenopus* epidermis, SEPT7 forms a ring at the base of motile cilia. Knockdown of SEPT7 leads to fewer and shortened cilia [23]. Although SEPT7 is important for ciliogenesis, it is still unclear whether post-translational modifications of SEPT7 affect ciliogenesis. Here we showed that SEPT7 phosphorylation was reduced during serum deprivation, and T197E mutant inhibited ciliogenesis, suggesting that the phosphorylation status of SEPT7 was important for the growth of primary cilia. So far, it is still unclear how T197E affects ciliogenesis. Septin filaments orchestrated the transition of primary cilia, and T197E mutants disrupted septin complex formation; thus, we speculate that the T197E mutant disrupts septin filament formation and inhibits ciliogenesis. However, this hypothesis still needs to be confirmed. In primary kidney inner medullary collecting duct (IMCD3) cells, blocking the expression of SEPT2 inhibits ciliogenesis, thus reducing Sonic hedgehog (Hh) signaling [22]. Hedgehog signaling provides an essential role in cilia function. Knockdown of SEPT7 causes defective ciliogenesis and abrogated Hedgehog signaling in developing *Xenopus* embryos [23]. Interestingly, PKA is a conserved negative regulator of the Hh signaling transduction. Increasing the cAMP concentration leads to the activation of PKA, then induces Gli3 repressor (Gli3R). Gli3R translocates to the nucleus and represses gene expression. PKA phosphorylated SEPT7, thus inhibiting ciliogenesis; thus, PKA can inhibit Hh signaling through activation of Gli3R or the disruption of primary cilia. However, this hypothesis still needs to be confirmed. Several studies have demonstrated that septin filament might play an exclusive role in membrane diffusion barrier functions in diverse cell types, including sperm, neurons, and epithelia. In the sperm, a septin ring structure (annulus) connects the midpiece and the principal piece. The SEPT4 null mice show a disruption of the diffusion barrier phenotype at the sperm tail [32]. In the epithelia, the absence of septin filaments caused the misalignment of microtubules, loss of apicobasal polarity [33,34], and an alteration of the cell shape [2], thus leading to defective cytokinesis [35,36,37]. In the nervous system, septins form diffusion barriers at the dendritic spine’s neck to restrict the diffusion of membrane proteins across the spine [38,39]. In this paper, we show that PKA-mediated SEPT7 phosphorylation is important for septin filament assembly and ciliogenesis. We speculate that the SEPT7 complex serves as a diffusion barrier at the ciliary base and participates in a variety of receptor signaling pathways. However, the downstream pathways of PKA and SEPT7 require further investigation. Our findings provide an important avenue to decipher the dynamics of SEPT complex assembly, as well as the physiological roles of the SEPT7 complex.

## 5. Conclusions

In summary, we found that SEPT7 was phosphorylated by PKA. PKA-mediated SEPT7 phosphorylation impeded SEPT7‒SEPT7 interaction, but did not affect the SEPT2-6-7 or SEPT4-6-7 complexes. In addition, phosphorylation of SEPT7 was reduced upon serum starvation, during which primary cilium started to grow. The T197E mutant inhibited ciliogenesis upon serum starvation. Thus, the phosphorylation status of SEPT7 is essential for septin filament formation and ciliogenesis.

## Data Availability

Not applicable.

## Supplementary Materials

The following are available online at https://www.mdpi.com/2073-4409/10/2/361/s1, Figure S1: Treatment with Na_3_VO_4_ does not change SEPT7 self-interaction.
