# Supplementary material for: Protein Kinase A-Mediated Septin7 Phosphorylation Disrupts Septin Filaments and Ciliogenesis
Source: Cells. 2021 Feb 9;10(2):361. doi: 10.3390/cells10020361 (PMC7916176; doi:10.3390/cells10020361)
Supplement: Supplementary file 1 [file cells-10-00361-s001.pdf]

Supplementary Figures

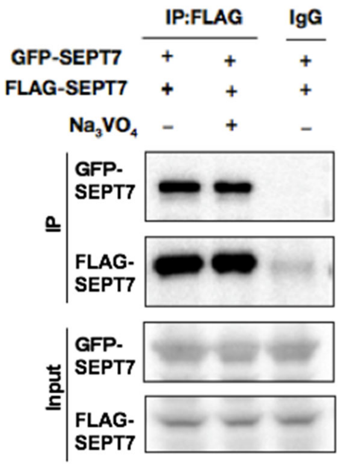

Figure S1. Treatment with Na_3VO_4 does not change SEPT7 self-interaction.
